# The Extract of Herbal Medicines Activates AMP-Activated Protein Kinase in Diet-Induced Obese Rats

**DOI:** 10.1155/2013/756025

**Published:** 2013-03-07

**Authors:** Hye-Yeon Shin, SaeYeon Chung, Soon Re Kim, Ji-Hye Lee, Hye-Sook Seo, Yong-Cheol Shin, Seong-Gyu Ko

**Affiliations:** ^1^Laboratory of Clinical Biology and Pharmacogenomics and Center for Clinical Research and Genomics, Institute of Oriental Medicine, Kyung Hee University, 26 Kyungheedae-ro, Dongdaemun-gu, Seoul 130-701, Republic of Korea; ^2^Department of Preventive Medicine, College of Oriental Medicine, Kyung Hee University, 26 Kyungheedae-ro, Dongdaemun-gu, Seoul 130-701, Republic of Korea

## Abstract

Our study investigated whether the extract of six herbal medicines (OB-1) has an inhibitory effect on obesity. High-fat diet-(HFD-) induced rats and controls were treated with 40 mg/100 g body weight of OB-1 or saline once a day for 5 weeks. After significant changes in body weight were induced, OB-1 and saline were administered to each subgroup of HFD and control groups for additional 5 weeks. No statistically significant decrease of body weight in OB-1-treated rats was found compared to controls. However, OB-1-treated rats were found to be more active in an open-field test and have a reduction in the size of adipocytes compared to controls. We observed no changes in the mRNA expressions of leptin and adiponectin from adipocytes between OB-1- and saline-treated rats with HFD-induced obesity group. However, OB-1 treatments were shown to be inversely correlated with accumulation of lipid droplets in liver tissue, suggesting that OB-1 could inhibit a lipid accumulation by blocking the pathway related to lipid metabolism. Moreover, the phosphorylation of AMP-activated protein kinase (AMPK) was significantly increased in OB-1-treated rats with HFD compared to controls. These results suggest that OB-1 has no direct antiobesity effect and, however, could be a regulator of cellular metabolism.

## 1. Introduction

Obesity due to disequilibrium of energy intake and expenditure has reached epidemic proportions in some parts of the world. Besides higher fat mass and body weight [1], obesity is associated with a higher risk for health problems such as cardiovascular disease, insulin resistance and diabetes mellitus, hyperlipidemia, arthrosis, many forms of cancer, and psychological stress [2, 3]. 

OB-1 consists of *Benincasae semen*, *Laminaria japonica* Areschon., *Pini Folium*, *Moli Folium*, *Citrus aurantium* Linn., and *Ephedra herb* (Materia medica, ISBN: 8985897373). *Benincasae semen* is a diuretic that has been used to eliminate toxins and edema from the body since early times. *Laminaria japonica Areschon* was reported to have an effect of anti-obesity [4]. It is known that *Pini Folium* increases serum lipid metabolism, and *Moli Folium* suppresses obesity. It was also reported that *Citrus aurantium Linn* increases the basal metabolic rate, acts as a diuretic, and reduces the activity of lipase [5]. The *Ephedra herb* is a well-known anti-obesity medicine that reduces body weights [6]. 

Obesity-induced alterations in adipocyte tissue result in altered expression or function of important endocrine hormones like leptin and adiponectin. Fasting leptin levels are remarkably elevated in adipocyte from obese individuals, and its gene expression is significantly increased in rats with diet-induced obesity [1, 7]. Unlike leptin, adiponectin is reduced in adipocyte tissue from obese individuals [8].

AMPK is known as a key molecule that regulates energy balance, body weight, food intake, and metabolic balance of lipid and glucose. The activation of AMPK switches cells from ATP consumption to active ATP-producing processes like fatty acid and glucose oxidation. From these reasons, AMPK has become the focus of many recent studies as a therapeutic target of metabolic disease [9–11].

## 2. Methods and Materials

### 2.1. Preparation of OB-1

Six herbs, *Benincasae semen, Laminaria japonica Areschon, Pini Folium, Moli Folium, Citrus aurantium Linn,* and* Ephedra herb,* were purchased from Omniherb (Gyeong Buk, Korea) and immersed in 1 L of 80% ethanol and then sonicated for 30 min. The resulting extract was filtered through a glass filter using a vacuum pump. A rotary vacuum evaporator (Eyela, Japan) was used to concentrate the liquid extract at 45°C. The concentrated extract was then lyophilized and reconstituted in saline at the working concentration. OB-1 is prepared from these six herbs extracts in the ratio of 1 : 1 : 1 : 1 : 1 : 1.

### 2.2. Experimental Design

Four-week-old male Wistar rats weighing 140–160 g were purchased from Central Laboratory Animal, Inc. (Seoul, Republic of Korea). The animals were examined in compliance with Guide for Animal Experiments edited by the Korean Academy of Medical Sciences. Four rats were housed per cage under a 12 : 12 hour light-dark cycle, 50% humidity, and 23 ± 1°C. The nutrient component and composition ratio of the control and high-fat diets are indicated in Table 1 [1, 12]. The rats were fed with a standard laboratory pellet chow (Purina Co.; Republic of Korea) and acclimatized to their environment for 7 days before commencing the experiment. After acclimatization, the control group (*n* = 8) received a standard laboratory chow diet (control diet) and the high-fat diet group (*n* = 10) received the diet described in Table 1. The nutrient component of the control diet (3.665 kcal/g) was 65% carbohydrate, 20% protein, and 4.5% lipid. The high-fat diet (4.058 kcal/g) was a mix containing highly palatable human foods (cookies, cheese, sausage, chips, chocolate, and almonds) in a proportion of 2 : 2 : 2 : 2 : 1 : 1 and an equal amount (in grams) of the control laboratory chow diet. This high-fat diet contained 32%, 12%, and 31% of its energy as carbohydrate, protein, and fat, respectively. The animals were weighed at the start of the experiment and every week thereafter. After 5 weeks of feeding the rats either control or high-fat diets, each group was randomly divided into saline-treated or OB-1-treated groups. Rats were fed the indicated diet treated with saline or 40 mg/100 g of OB-1 daily for 5 weeks. Rats were sacrificed by administration of anesthesia 10 weeks after the start of the dietary treatment. 

### 2.3. Organ Samples

Epididymal adipose tissue and liver samples were enucleated from the rats and washed in cold saline solution. Epididymal adipocyte samples were immediately stored in a −70°C deep freezer for subsequent mRNA isolation. Liver samples were fixed overnight in 10% neutral buffer formalin (NBF) in preparation for histological staining. Fixed liver samples were then soaked in 30% sucrose (Sigma; St. Louis, MO, USA) until the liver samples sank to the bottom of bottle. After removing excess fluid from samples, they were stored at −70°C.

### 2.4. RT-PCR Analysis

Total RNA was isolated using TRIzol Reagent according to the manufacturer's instructions (Invitrogen; Grand Island, NY, USA). Total RNA was treated with 2 units of RNase-free DNase (Promega, Madison, WI, USA) at 37°C for 30 min, extracted with phenol/chloroform/isopropanol (25 : 24 : 1; Fluka; Milwaukee, Wi, USA), and precipitated with ethanol. For reverse transcriptase (RT) reactions, 2 *μ*g of total RNA was used as a template to synthesize cDNA, as follows: total RNA was combined with 4 *μ*g random hexamer (Amersham Biosciences; Buckinghamshire, UK), incubated at 65°C for 10 min, and cooled on ice for 2 min. The RT reaction was carried out in a 30-*μ*L total volume with 2 units of M-MLV reverse transcriptase (Invitrogen; Carlsbad, CA, USA) at 42°C for 1 h, followed by heating at 95°C for 5 min to stop the reaction. Subsequent PCR was carried out in a 25-*μ*L reaction mixture consisting of the cDNA template, 10 pmole of each gene-specific primer, 5X first strand buffer, 2.5 mM dNTP mixture, and 1 unit of* Taq* DNA polymerase (Takara Korea; Seoul, Republic of Korea). PCR was performed using the following primers for leptin (5′ ATG TGC TGG AGA CCC CTG T 3′; 5′ ATT CAG GGC TAA GGT CCA ACT 3′) and GAPDH (5′ CAA AGT GGA CAT TGT TGC CA 3′; 5′ TTC ACC ACC TTC TTG ATG TCA 3′). The resulting PCR products were resolved in 2.0% agarose gels containing ethidium bromide.

### 2.5. Immunoblot Analysis

Tissues were homogenized in buffer containing 50 mM Tris-HCl, pH 7.4, 5 mM EDTA, 150 mM NaCl, 0.1% sodium dodecyl sulfate (SDS), 1% Triton X-100, and protease inhibitor cocktail (104 mM AEBSF, 0.08 mM Aprotinin, 2 mM Leupeptin, 4 mM Bestatin, 1.5 mM Pepstatin A, and 1.4 mM E-64) on ice. Homogenized tissue was incubated for 30 min on ice, followed by centrifugation at 14,000 rpm for 30 min at 4°C. The supernatant was used to conduct a Bradford assay (Bio-rad) to determine protein concentration. Then, 50 *μ*g of total protein was separated on 10% reducing polyacrylamide gels and transferred to membranes. Immunoblot analysis was performed using a phospho-AMPK antibody (Cell Signaling Technology; Beverly, MA, USA) or *α*-tubulin antibody, and immunoreactive proteins were detected using chemiluminescence. 

### 2.6. Isolation of Fat Cell from Adipocyte

Fat cells were isolated by collagenase treatment, in accordance with a method previously described [13]. Briefly, epididymal adipose tissue samples were minced at room temperature and incubated with 1.5 g/L of collagenase in 10 mL Krebs-Ringer bicarbonate (KRB; 10 nM HEPES, 6 mM glucose, and 30 g/L bovine serum albumin, pH 7.4, pregassed with 95% O_2_/5% CO_2_) for 30 min at 37°C in a shaking water bath. Adipocytes were then visualized by microscopy and photographed.

### 2.7. Liver Morphology

The fixed liver samples described above were embedded in Optical Cutting Temperature (OCT) compound, and 10 *μ*M sections were cut on a cryostat. Tissue sections were stained with Oil Red O (Sigma), to visualize neutral lipids, and nuclei were counterstained with hematoxylin (Gill No. 2; Sigma). Oil Red O was dissolved in 99% isopropanol, left overnight at room temperature, and filtered with Whatman filter paper no. 2 (Whatman; UK). This stock solution was mixed with distilled water (2 : 3) and refiltered with Whatman filter paper no. 2 before use. Slides containing sectioned liver tissue were rinsed with isopropanol for 10 min and stained with the Oil Red O working solution for 15 min. The slide was then decolorized with 70% isopropanol for 3 min, rinsed with distilled water for 5 min, and stained with hematoxylin for 30 sec. Stained slides were given a final wash with distilled water, air dried, and mounted with glycerin jelly. 

### 2.8. Open Field Test

To determine whether OB-1 treatment increased the activity level of rats, we monitored and compared visit counts, rearing, and grooming of the OB-1-treated group to the saline group in an open field arena. We used a 75 cm × 75 cm arena with 30 cm high walls, constructed of five pieces of thick paper and marked with a cross stripe across the bottom at a distance of 15 cm. A video camera was placed 250 cm above the arena, and it was used to record 10 min of activity per rat. Incidences of three kinds of activity (visit counts, rearing, and grooming) were monitored. 

### 2.9. Statistical Analysis

Statistical analyses were performed using window SPSS (version 12.0). All of the quantitative data were analyzed by independent *T*-tests for the differences between two means and one-way ANOVA for the differences among four means. *P* values of < 0.05 indicated significant differences.

## 3. Results

### 3.1. Effect of OB-1 on Body Weight of High-Fat Diet-Induced Rats

**Figure 1:**
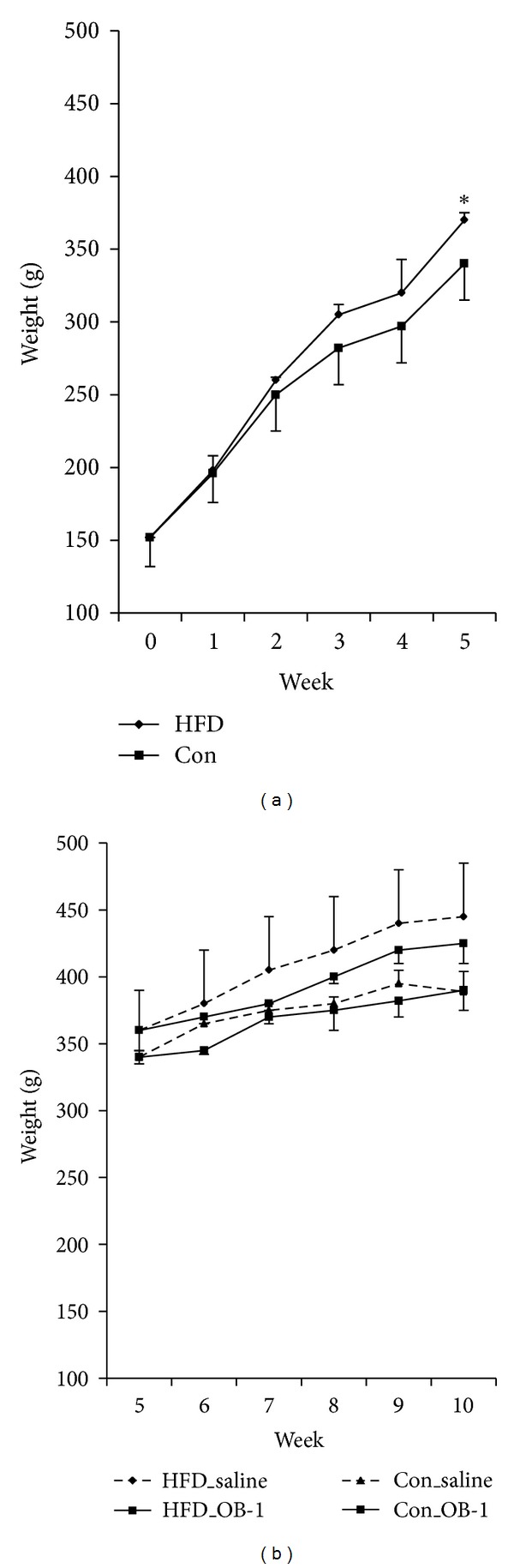
The change of body weight for total 10 weeks. (a) Obesity in rats (*n* = 10) was induced by high-fat diet for five weeks compared to controls (*n* = 8). (b) After subclassification of each group, herbal medicine (OB-1) and saline were treated for additional five weeks. Each data presents the mean ± S.E.M. *P < 0.05 compared to controls.

High-fat diet-induced rats (*n* = 10) were significantly overweighted compared to controls of standard laboratory chow diet rats (*n* = 8) after five weeks (*P* = 0.02, Figure 1). Then, half of each HFD-induced and control groups were randomly subclassified and administered with OB-1 and saline for additional five weeks, as like HFD_OB-1, HFD_saline, Con_OB-1, and Con_saline, respectively. OB-1 treatments in HFD-induced rats showed about 3.1% reduction of body weight at 10 weeks, but not significant (*P* > 0.05). In addition, there was no difference in food intake between OB-1 and saline treatment groups. As like HFD group, there was no difference between OB-1 and saline treatment of controls in body weight.

### 3.2. Increased Activity of Movements in Open-Field Test by OB-1

**Figure 2:**
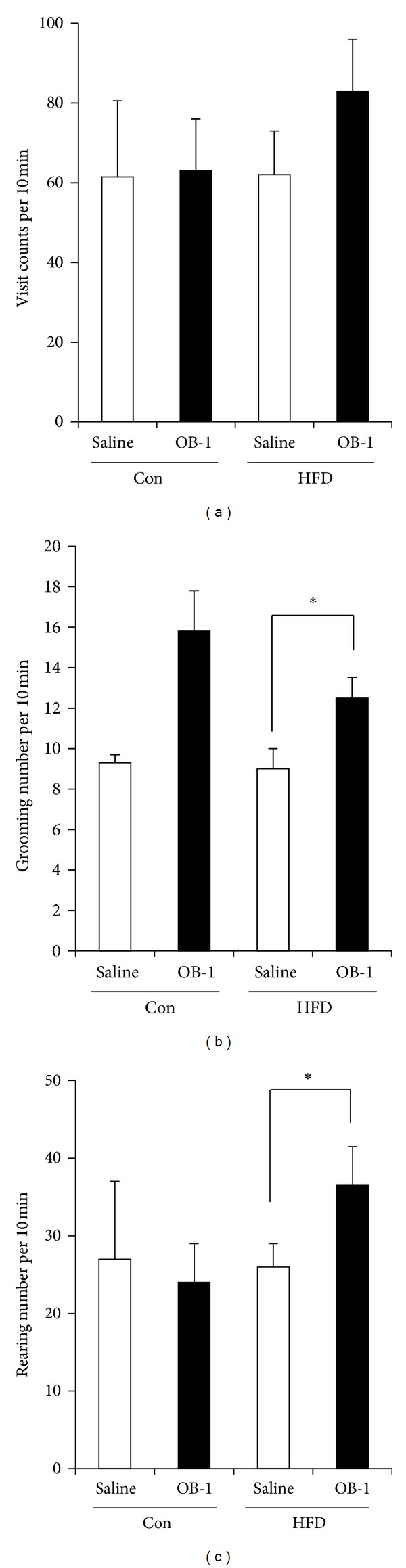
Increased activity of movements in open field test by OB-1. Animals were placed in open field arenas and allowed to move freely for 10 minutes. Visit counts (a), grooming (b), and rearing (c) of control_saline (*n* = 4), control_OB-1 (*n* = 4), HFD_saline (*n* = 5), HFD_OB-1 (*n* = 5) were monitored to measure activity levels. Each data presents the mean ± S.E.M. *P < 0.05 compared to saline treatment of HFD-induced obese rats.

To evaluate the effects of OB-1 on general activity levels of rats, rats of each group were subjected to an open field test to evaluate their levels of activity. During the 10 min they were allowed to roam freely, the visiting, rearing, and grooming of rats were monitored. Interestingly, OB-1 treatments in HFD-induced rats significantly increased the activity of movements compared to HFD_saline group (*P* < 0.05, Figure 2). 

### 3.3. Effects of OB-1 on Adipocytes and Lipid Accumulation

**Figure 3:**
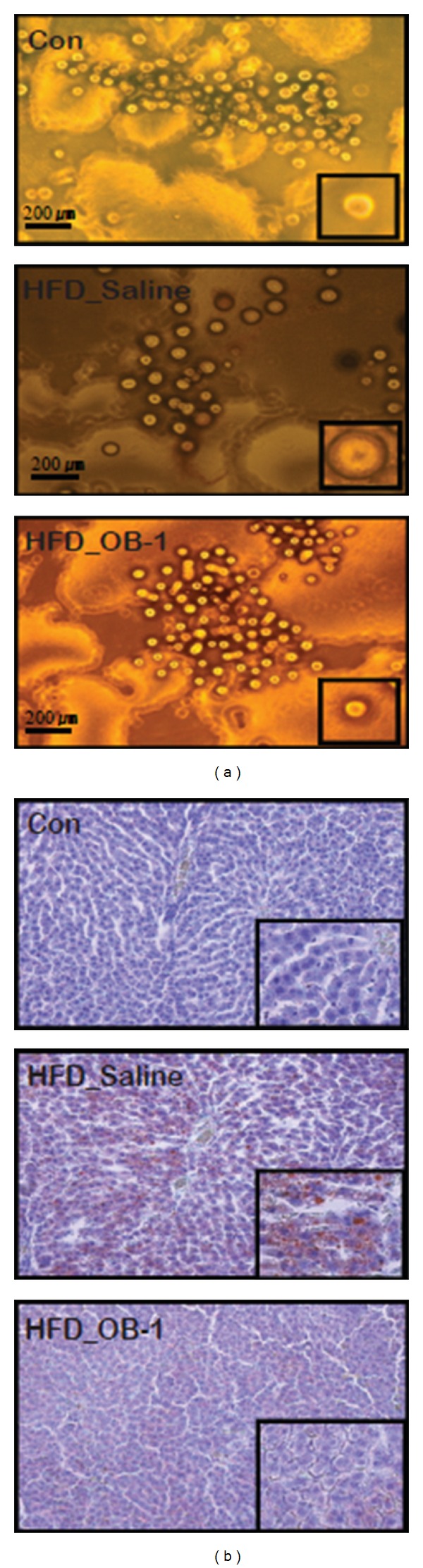
Effects of OB-1 on adipocytes and lipid accumulation. (a) Photomicrographs of isolated epididymal white adipocytes from HFD-induced rats. (b) For microscopic observation (200x), liver tissues from each group were stained with Oil Red O to visualize neutral lipids and counterstained the nuclei with hematoxylin.

To observe whether OB-1 affects the morphology of cells, white adipocytes cells were first isolated from rats of each group and then compared by microscopy. Epididymal white adipocytes from HFD-induced rats were shown to be enlarged compared to the controls that were fed standard chow diet, whereas the size of adipocytes from OB-1-administered rats with HFD-induced obesity was found to be recovered (Figure 3(a)). Since obesity is related to lipid accumulation in the liver, liver tissues from each group were harvested and then stained with Oil Red O, a dye specific for lipid staining, and counterstained the nuclei with hematoxylin. Microscopic observation showed lipid droplets in the liver tissues from obesity-induced rats without administration of OB-1 (HFD_saline), whereas those from OB-1 treatment group showed no lipid droplets, similar to control samples (Figure 3(b)), indicating that OB-1 treatments could modulate the formation of lipid droplets in liver tissues. 

### 3.4. Effects of OB-1 on the Expression of Leptin and Adiponectin mRNA

**Figure 4:**
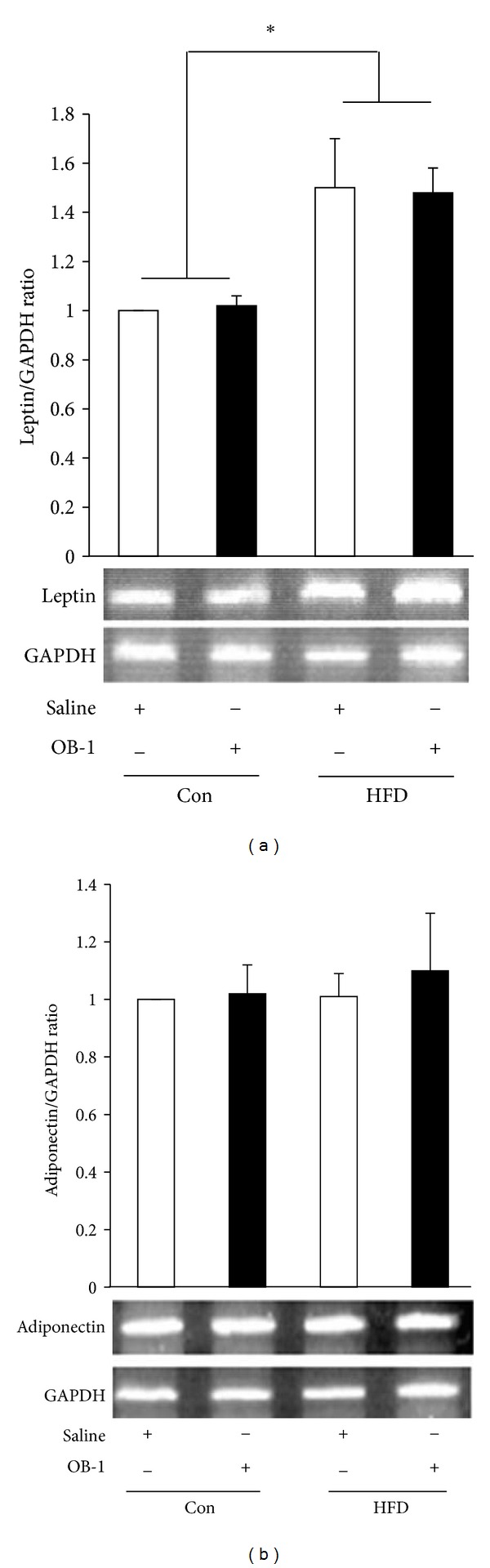
No change in mRNA expressions of leptin and adiponectin. Transcriptional levels of leptin (a) and adiponectin (b) were determined by RT-PCR to evaluate the effect of OB-1 on HFD-induced obese rats. Each data presents the mean ± S.E.M. from three independent experiments. *P < 0.05 compared between HFD-induced rats and controls.

Based on the changes in adipocytes and liver tissue after OB-1 treatments, it was investigated whether the expression of leptin and adiponectin genes could be regulated by OB-1. Although the transcriptional level of leptin in epididymal white adipocytes from HFD-induced rats was significantly increased compared to controls, there was no difference between OB-1 and saline treatments (Figure 4(a)). In case of adiponectin, each group showed no significant change (Figure 4(b)). These results indicate that OB-1 has no effect to regulate the expression of both leptin and adiponectin. 

### 3.5. Stimulatory Effect of OB-1 on the Phosphorylation of AMPK

**Figure 5:**
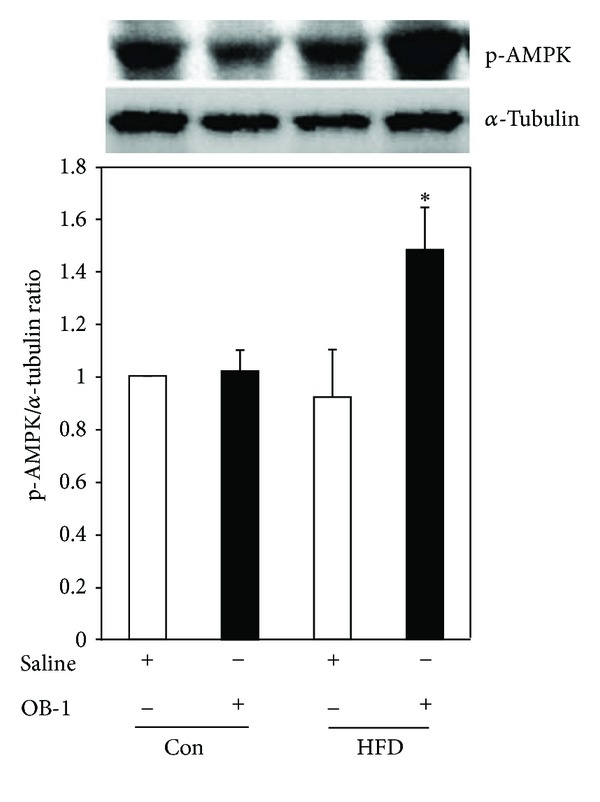
Stimulatory effect of OB-1 on the phosphorylation of AMPK. The phosphorylated AMPK protein levels in liver tissue were detected by western blot analysis. OB-1 treatment to HFD-induced obese rats showed a significant increase in phosphorylation of AMPK compared to saline treatment. Each data presents the mean ± S.E.M. from three independent experiments. *P < 0.05 compared to saline treatment of HFD-induced obese rats.

Since AMPK plays a key role in energy metabolism within cells, it was further investigated whether OB-1 affects AMPK activity in the liver tissues from rats. There were no differences in the AMPK protein expression among controls and saline-administered group with HFD-induced obesity. However, the phosphorylation of AMPK was significantly increased in the OB-1-administered group compared to saline-treated obese group as well as controls (*P* < 0.05, Figure 5). 

## 4. Discussion

OB-1, a mixture of six herbal remedies, has been utilized for detoxification or metabolic applications in oriental medicine. Each of the six components (*Benincasae semen*, *Laminaria japonica Areschon*, *Pini Folium*, *Moli Folium*, *Citrus aurantum Linn,* and *Ephedra herb*) has been independently reported to exert its clinical effects on the human body, including regulation of lipid metabolism, reduction of body fat mass, and elimination of toxic compounds. *Citrus aurantum*, as a dietary supplement to reduce obesity, was examined to promote weight loss and metabolic rate [14, 15]. The *Ephedra herb* is a well-known anti-obesity medicine for weight loss. Multinutrient containing *ephedra* especially has been shown to have effects of weight loss and to improve metabolic risks in obese persons [16]. The* Laminaria japonica* is widely eaten as a healthy food in East Asia, and its preventive effects in streptozotocin-induced diabetic rat liver were reported recently [17]. Therefore, we hypothesized that OB-1 might have a significant effect on obesity and then established and evaluated its molecular functions using *in vitro* and *in vivo* studies. To induce an obesity (significant changes in body weight), 5-week treatment with high-fat diet was appropriate in our experimental model. Moreover, 40 mg/100 g body weight of OB-1 dose and additional 5-week treatments were appropriate to observe activity of movements in open field test, reduction in the size of adipocytes, inhibition of lipid accumulation, and phosphorylation of AMPK. Our findings revealed that OB-1 had no direct effect on anti-obesity and regulation of leptin and adiponectin; however, OB-1 increased the phosphorylation of AMPK and the activity of movement *in vivo*. 

AMPK plays an important function in maintaining the energy balance within cells. Also, liver is a center for energy metabolism and glucose homeostasis. To control energy homeostasis, AMPK activation decreases gluconeogenesis and lipogenesis, whereas it increases fat oxidation and glucose uptake by switching on ATP-generating pathways and switching off ATP-consumption pathways [9, 18, 19]. In this study, OB-1 treatments were also shown to be negatively correlated with accumulation of lipid droplets in liver tissue, suggesting that OB-1 could inhibit a lipid accumulation by blocking the pathway related to lipid metabolism. In support of this idea, OB-1 positively regulated the AMPK activity in liver tissue from OB-1-treated group with HFD-induced obesity. Therefore, OB-1 might negatively regulate the lipid metabolism or gluconeogenesis by activating AMPK. However, it is needed to elucidate the underlying molecular mechanisms in detail. 

According to our results of open field tests [20], OB-1-administered rats with HFD-induced obesity showed the increased grooming and rearing activities compared to saline-treated control group. Although OB-1-administered obese rats showed no significant reduction of body weight compared to saline-treated obese group, these increased movements suggest that the activation of AMPK and its related mechanisms might control not body weight or food intake but energy metabolism [10, 21]. This increased energy metabolism by OB-1 could decrease about 3.1% reduction of body weight of rats in this study, but not significant, and might increase the activity of movements. 

Leptin and adiponectin, as adipocyte-derived hormones, play key roles in obesity and energy homeostasis [22, 23]. Since leptin regulates body fat stores through its effects on food intake and energy metabolism, leptin is an important molecule in the obesity process [24]. It has been recently reported that there is a significant correlation between leptin expression and adipocyte size [25]. In addition, there is a correlation between the expression of adiponectin, which is an adipokine that is specially secreted by adipocytes, and adipocyte size in obesity [26, 27]. Based on these facts, the expression of leptin and adiponectin was evaluated, but no significant change in the mRNA expression of the genes by treatment of OB-1 was found. However, the size of adipocyte from OB-1-administered rats with HFD-induced obesity was shown to be recovered compared to the enlarged adipocytes of obesity-induced rats, suggesting that other signals might be involved in the expression and regulation of adipocytes in treatment of OB-1. It was reported that TNF is expressed in human adipocytes, and TNF level is positively correlated with obesity [28]. This suggests that TNF signaling might be involved in the expression and regulation of adipocytes in treatment of OB-1.

Physical exercise and contraction are correlated with the increased phosphorylation of AMPK in human or rat skeletal muscle [29, 30]. Therefore, our findings that administration of OB-1 increased the phosphorylation of AMPK suggest that components from the six herbals could stimulate the mechanism of physical movements and exercise through the activation of AMPK and/or its involved regulators. In conclusion, this study showed that treatments with OB-1 to HFD-induced obese rats significantly increased the phosphorylation of AMPK and reduced the enlarged size of adipocytes from HFD-induced obesity and lipid accumulation. Therefore, it is suggested that these effects of OB-1, especially related with the activation of AMPK, might alter the metabolic processes. Our findings need future studies at the levels of molecular mechanism to understand how OB-1 herbal extract or its components modulate metabolic processes.

## Figures and Tables

**Table 1:** The nutrient component and composition ratio of control and high-fat diet.

Component	Experimental diet (g/kg diet)	
	Control diet	High-fat diet
Protein	200.0	120.0
Carbohydrate	615.0	320.0
Fat	45.0	310.0
Fiber	60.0	30.0
Crude ash	70.0	35.0
Alsium	5.0	2.5
Phosphorous	5.0	2.5

Energy (kcal/g)	3.665	4.058

## References

[1] Pérez-Echarri N, Pérez-Matute P, Martínez JA, Marti A, Moreno-Aliaga MJ (2005). Serum and gene expression levels of leptin and adiponectin in rats susceptible or resistant to diet-induced obesity. *Journal of Physiology and Biochemistry*.

[2] Kahn BB, Flier JS (2000). Obesity and insulin resistance. *Journal of Clinical Investigation*.

[3] Karlsson J, Taft C, Sjöström L, Torgerson JS, Sullivan M (2003). Psychosocial functioning in the obese before and after weight reduction: construct validity and responsiveness of the Obesity-related Problems scale. *International Journal of Obesity*.

[4] Wang W, Wang WX, Sun BH, Zhao DZ, Gao P (2000). Effect of haidonghua powder(HDHP) on hypothalamic obesity in rats. *Zhongguo zhong yao za zhi*.

[5] Tsujita T, Sumiyoshi M, Han LK, Fujiwara T, Tsujita J, Okuda H (2003). Inhibition of lipase activities by citrus pectin. *Journal of Nutritional Science and Vitaminology*.

[6] Shekelle PG, Hardy ML, Morton SC (2003). Efficacy and safety of ephedra and ephedrine for weight loss and athletic performance: a meta-analysis. *Journal of the American Medical Association*.

[7] Lönnqvist F, Nordfors L, Jansson M, Thörne A, Schalling M, Arner P (1997). Leptin secretion from adipose tissue in women: relationship to plasma levels and gene expression. *Journal of Clinical Investigation*.

[8] Kern PA, di Gregorio GB, Lu T, Rassouli N, Ranganathan G (2003). Adiponectin expression from human adipose tissue: relation to obesity, insulin resistance, and tumor necrosis factor-*α* expression. *Diabetes*.

[9] Carling D (2004). The AMP-activated protein kinase cascade—a unifying system for energy control. *Trends in Biochemical Sciences*.

[10] Kahn BB, Alquier T, Carling D, Hardie DG (2005). AMP-activated protein kinase: ancient energy gauge provides clues to modern understanding of metabolism. *Cell Metabolism*.

[11] Eun JK, Jung S-N, Kun HS (2007). Antidiabetes and antiobesity effect of cryptotanshinone via activation of AMP-activated protein kinase. *Molecular Pharmacology*.

[12] Darimont C, Turini M, Epitaux M (2004). *β*3-adrenoceptor agonist prevents alterations of muscle diacylglycerol and adipose tissue phospholipids induced by a cafeteria diet. *Nutrition and Metabolism*.

[13] Llado I, Estrany ME, Rodriguez E, Amengual B, Roca P, Palou A (2000). Effects of cafeteria diet feeding on *β*3-adrenoceptor expression and lipolytic activity in white adipose tissue of male and female rats. *International Journal of Obesity*.

[14] Bent S, Padula A, Neuhaus J (2004). Safety and efficacy of citrus aurantium for weight loss. *The American Journal of Cardiology*.

[15] Haaz S, Fontaine KR, Cutter G, Limdi N, Perumean-Chaney S, Allison DB (2006). Citrus aurantium and synephrine alkaloids in the treatment of overweight and obesity: an update. *Obesity Reviews*.

[16] Hackman RM, Havel PJ, Schwartz HJ (2006). Multinutrient supplement containing ephedra and caffeine causes weight loss and improves metabolic risk factors in obese women: a randomized controlled trial. *International Journal of Obesity*.

[17] Jin DQ, Li G, Kim JS, Yong CS, Kim JA, Huh K (2004). Preventive effects of Laminaria japonica aqueous extract on the oxidative stress and xanthine oxidase activity in streptozotocin-induced diabetic rat liver. *Biological and Pharmaceutical Bulletin*.

[18] Hardie DG, Scott JW, Pan DA, Hudson ER (2003). Management of cellular energy by the AMP-activated protein kinase system. *FEBS Letters*.

[19] Zhou G, Sebhat IK, Zhang BB (2009). AMPK activators—potential therapeutics for metabolic and other diseases. *Acta Physiologica*.

[20] Grzeda E, Wiśniewska RJ, Wiśniewski K (2007). Effect of an NMDA receptor agonist on T-maze and passive avoidance test in 12-week streptozotocin-induced diabetic rats. *Pharmacological Reports*.

[21] Viollet B, Guigas B, Leclerc J (2009). AMP-activated protein kinase in the regulation of hepatic energy metabolism: from physiology to therapeutic perspectives. *Acta Physiologica*.

[22] Ahima RS, Flier JS (2000). Leptin. *Annual Review of Physiology*.

[23] Yamauchi T, Kamon J, Waki H (2001). The fat-derived hormone adiponectin reverses insulin resistance associated with both lipoatrophy and obesity. *Nature Medicine*.

[24] Caro JF, Sinha MK, Kolaczynski JW, Zhang PL, Considine RV (1996). Leptin: the tale of an obesity gene. *Diabetes*.

[25] Kim SO, Yun SJ, Jung B (2004). Hypolipidemic effects of crude extract of adlay seed (*Coix lachrymajobi var. mayuen*) in obesity rat fed high fat diet:Relations of TNF-*α* and leptin mRNA expressions and serum lipid levels. *Life Sciences*.

[26] Arita Y, Kihara S, Ouchi N (1999). Paradoxical decrease of an adipose-specific protein, adiponectin, in obesity. *Biochemical and Biophysical Research Communications*.

[27] Bauche IB, El Mkadem SA, Pottier AM (2007). Overexpression of adiponectin targeted to adipose tissue in transgenic mice: Impaired adipocyte differentiation. *Endocrinology*.

[28] Kern PA, Saghizadeh M, Ong JM, Bosch RJ, Deem R, Simsolo RB (1995). The expression of tumor necrosis factor in human adipose tissue. Regulation by obesity, weight loss, and relationship to lipoprotein lipase. *Journal of Clinical Investigation*.

[29] Fujii N, Hayashi T, Hirshman MF (2000). Exercise induces isoform-specific increase in 5’ AMP-activated protein kinase activity in human skeletal muscle. *Biochemical and Biophysical Research Communications*.

[30] Wojtaszewski JFP, Nielsen P, Hansen BF, Richter EA, Kiens B (2000). Isoform-specific and exercise intensity-dependent activation of 5’-AMP-activated protein kinase in human skeletal muscle. *Journal of Physiology*.

